# Survey of Adrenal Insufficiency Management for Duchenne muscular dystrophy in Italy

**DOI:** 10.1007/s12020-026-04596-6

**Published:** 2026-04-01

**Authors:** Gianluca Tornese, Sze Choong Wong, Anne Marie Sbrocchi, Fernanda De Angelis, Ilaria Zito, David R. Weber, Tommaso Aversa

**Affiliations:** 1 Department of Medicine, Surgery and Health Sciences, University of Trieste, Trieste, Italy; 2 Institute for Maternal and Child Health IRCCS “Burlo Garofolo”, Trieste, Italy; 3 Department of Paediatric Endocrinology, Royal Hospital for Children, Glasgow, UK; 4 Bone, Endocrine, Nutrition Research Group in Glasgow, University of Glasgow, Glasgow, UK; 5 Division of Pediatric Endocrinology and Metabolism, Montreal Children’s Hospital, McGill University Health Centre, Montreal, Canada; 6 Shriners Hospital for Children, Montreal, Canada; 7 Parent Project aps, Rome, Italy; 8 Division of Endocrinology and Diabetes, The Children’s Hospital of Philadelphia and Perelman School of Medicine at the University of Pennsylvania, Philadelphia, USA; 9 Department of Human Pathology of Adulthood and Childhood, University of Messina, Messina, Italy; 10 Pediatric Unit, ”G. Martino” University Hospital, Messina, Italy

**Keywords:** Duchenne muscular dystrophy, adrenal insufficiency, steroid treatment, management, education, Italy

## Abstract

**Purpose:**

The aim of this study was to evaluate current real-world practices for the prevention and management of adrenal insufficiency (AI) among Italian physicians caring for children and adolescents with Duchenne muscular dystrophy (DMD) receiving chronic glucocorticoid (GC) therapy, a treatment known to improve motor and cardiopulmonary outcomes but to suppress the hypothalamic–pituitary–adrenal axis and increase the risk of adrenal crisis.

**Methods:**

A cross-sectional online survey was distributed via national networks to Italian pediatric endocrinologists and neuromuscular specialists. The questionnaire explored clinician characteristics, patient caseloads, glucocorticoid regimens, stress-dose recommendations for mild, moderate, and severe illness, and proactive measures such as family education, emergency kits, written plans, medical alert identification, and electronic hospital alerts. Participation was anonymous.

**Results:**

Thirty-five clinicians responded (57% pediatric endocrinologists, 43% neuromuscular specialists), most following 11–50 patients. Deflazacort was the predominant treatment (86%). For mild stress, 80% advised no additional GC. For moderate stress, 43% recommended no change to management, 31% provided oral hydrocortisone (HC), 17% advised extra daily GC, and 9% prescribed intramuscular HC. For severe stress, although 77% prescribed parenteral HC, 14% advised no change. Proactive measures were inconsistently implemented: 82% provided family education, 43% gave intramuscular HC prescriptions and training, 71% had written emergency plans, and 31% recommended medical alert identification.

**Conclusions:**

This national survey reveals substantial variability and gaps in AI prevention and management for children and adolescents with DMD on chronic GC therapy in Italy. Harmonized national protocols, multidisciplinary coordination, and strengthened family education are needed to improve adrenal crisis prevention.

**Supplementary Information:**

The online version contains supplementary material available at 10.1007/s12020-026-04596-6.

## Introduction

Duchenne muscular dystrophy (DMD), the most common inherited neuromuscular disorder of childhood (15.9–19.5 per 100 000 live male births), is characterized by early onset, progressive muscle degeneration, and early loss of ambulation, typically within the first decade. Without disease-modifying therapies, affected boys historically developed severe disability and premature death in the second decade due to respiratory or cardiac failure [1]. Long-term systemic glucocorticoid (GC) therapy has become a cornerstone of care, improving motor, respiratory, and cardiac outcomes and prolonging survival, though at the cost of multiple adverse effects [1–7].

Chronic exposure to supraphysiologic GC doses (usually > 8–10 mg/m² hydrocortisone [HC] equivalent) for longer than 3–4 weeks suppresses the hypothalamic–pituitary–adrenal (HPA) axis, resulting in secondary or tertiary adrenal insufficiency (AI) [8–13]. This suppression may persist months or years after GC withdrawal. The risk is greatest with daily regimens using prednisone/prednisolone (PN/PDL), deflazacort (DFZ), or vamorolone (VAM) [14–15], while data for intermittent and alternate-day schedules remain scarce. In children with DMD, AI is usually asymptomatic until stress, infection, surgery, or abrupt GC cessation triggers an adrenal crisis [16–19]. Current international guidelines emphasize caregiver education, stress-dose coverage, and emergency access to injectable HC [1, 20–23]. Nevertheless, real-world adherence remains inconsistent; patient preparedness and family education are often incomplete [24].

This study aimed to describe AI prevention and management practices among Italian physicians caring for children and adolescents with DMD on chronic GC therapy, hypothesizing marked intercenter and interspecialty variability and gaps relative to guideline recommendations.

## Materials and methods

### Study design and ethics

This national, cross-sectional survey targeted clinicians managing children and adolescents with DMD receiving GC therapy. Because only anonymized clinician-level data were collected, the study was exempt from formal institutional review board approval and informed consent requirements.

### Questionnaire

A 15-item online questionnaire (Google Form) was developed by pediatric endocrinologists experienced in DMD and AI. It explored:

1. Clinician and center characteristics (specialty, setting, and DMD caseload).
2. GC regimens (drug, dose pattern).
3. Stress-dose strategies for mild, moderate, and severe illness.
4. AI prevention measures: family education, emergency HC kits, written emergency plans, use of medical IDs, and electronic hospital alerts.

### Study population

Pediatric endocrinologists and neuromuscular specialists directly involved in prescribing or monitoring long-term glucocorticoid therapy from all 31 Italian DMD centers (5 pediatric hospitals, 26 mixed pediatric–adult) were invited through national professional networks [25]. In the Italian context, these clinicians typically include pediatricians subspecialized in endocrinology and child neuropsychiatrists or neurologists who practice primarily in pediatric settings. Participation was voluntary and anonymous.

### Analysis

Responses were exported into Microsoft Excel, cleaned, and coded for descriptive statistics. Categorical data were reported as counts and percentages. Subgroup analyses compared pediatric endocrinologists vs. neuromuscular specialists and smaller (≤ 50 patients) vs. larger (> 50 patients) centers using Fisher’s exact test (Jamovi v2.3.28). Statistical significance was defined as *p* < 0.05, without correction for multiple comparisons given the study’s exploratory intent.

## Results

### Respondents and centers

Thirty-five clinicians completed the survey, including 20 pediatric endocrinologists (57%) and 15 pediatric neuromuscular specialists (43%). The overall response rate was 64.5% (20/31) among invited pediatric endocrinologists and 48.4% (15/31) among invited pediatric neuromuscular specialists. Most respondents (57%) followed between 11 and 50 patients with DMD (65% of pediatric endocrinologists, 47% of pediatric neuromuscular specialists), while smaller proportions managed fewer than 5 patients (6%), 5–10 patients (14%), 51–100 patients (20%), or more than 100 patients (3%), with no statistically significant differences across specialties. A summary of clinician and center characteristics is provided in Table 1.

**Table 1:** Characteristics of survey respondents and participating centers**.** Values are expressed as number (percentage) of respondents. For specialty and practice setting, percentages are calculated over the 31 participating centers; for patient caseload and glucocorticoid (GC) regimens, percentages are calculated over the 35 individual clinicians. *Multiple responses were allowed for “most frequently prescribed GC regimens”. Abbreviations: DFZ, deflazacort; DMD, Duchenne muscular dystrophy; GC, glucocorticoid; PN, prednisone; PDN, prednisolone

Variable	Category	*n* (%)
*Specialty of respondent*	Pediatric endocrinologist	20 (57)
	Neuromuscular specialist	15 (43)
*Practice setting*	Pediatric hospital	5 (14)
	Mixed pediatric–adult hospital	26 (74)
*Patients with DMD followed per respondent*	< 5	2 (6)
	5–10	5 (14)
	11–50	20 (57)
	51–100	7 (20)
	> 100	1 (3)
Most frequently prescribed GC regimens*	Daily DFZ	30/35 (86)
	Daily PN/PDN	9/35 (26)
	Intermittent DFZ	5/35 (14)
	Intermittent PN/PDN	1/35 (3)

### Glucocorticoid regimens

Regarding glucocorticoid regimens, daily DFZ was the most frequently prescribed (86%), followed by daily PN/PDL in 9/35 (26%), intermittent DFZ in 5/35 (14%), and intermittent PN/PDL in 1/35 (3%), with comparable distributions across specialties and center sizes (Table 1).

### Stress-dose management

**Fig. 1:**
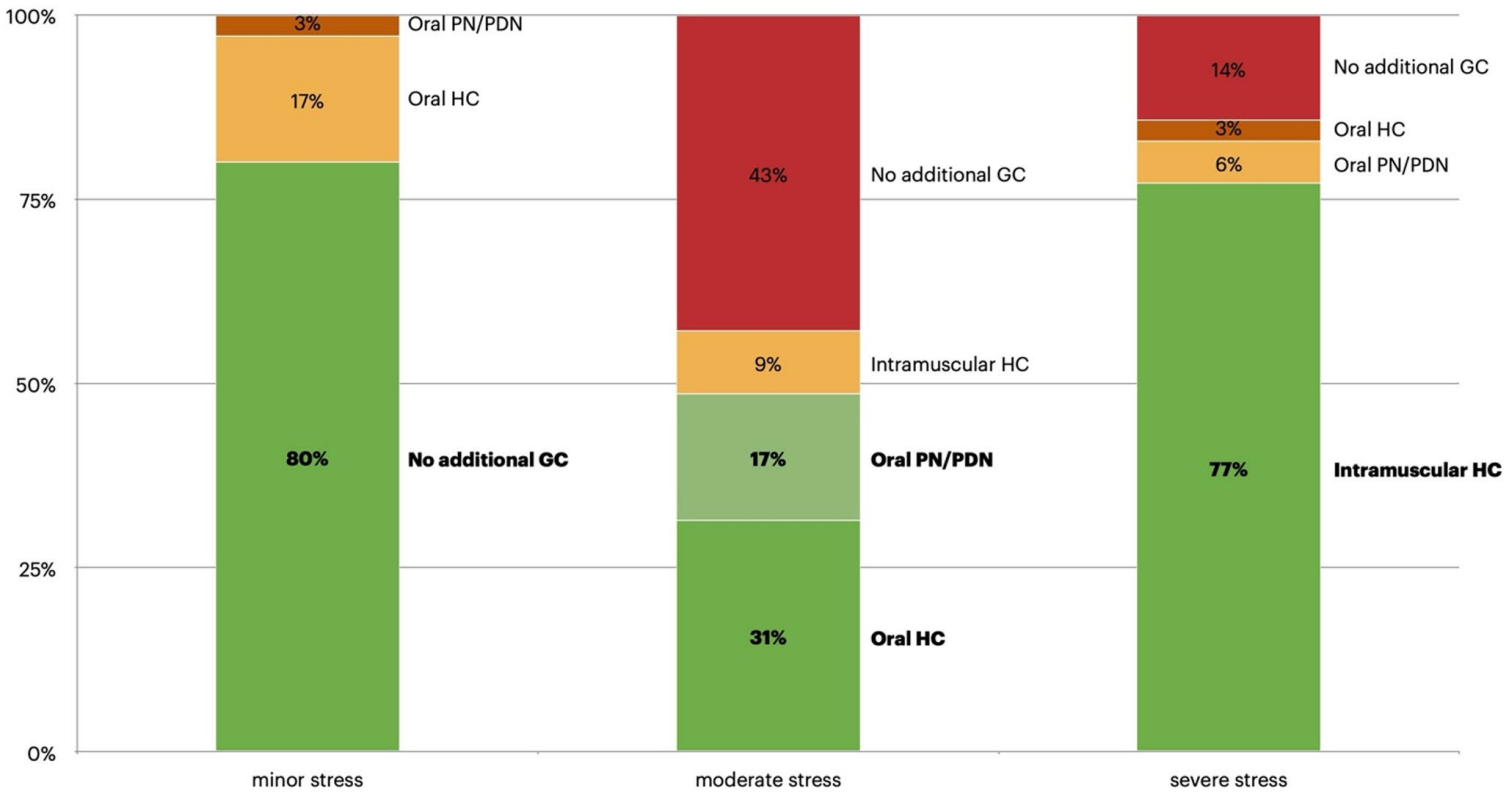
Management Approaches for Adrenal Insufficiency During Stress in Children and Adolescents With Duchenne Muscular Dystrophy on Glucocorticoid Therapy. Distribution of reported glucocorticoid adjustment strategies during minor, moderate, and severe stress. Bars represent the percentage of respondents recommending each strategy. Green indicates recommended or appropriate management; orange represents non-optimal but not harmful practices; red highlights potentially harmful or inappropriate management choices. Abbreviations: GC, glucocorticoid; HC, hydrocortisone; PN/PDL, prednisone/prednisolone

Reported stress-dose practices varied by both clinical scenario and specialty (Fig. 1):

- for *minor stress* (e.g. mild afebrile infections with preserved activity), 80% of clinicians recommended no additional GC; this approach was more frequent among pediatric neuromuscular specialists than pediatric endocrinologists (93% vs. 70%; *p* = 0.027) and among clinicians in high-caseload centers (≥ 50 patients) compared with smaller centers (100% vs. 76%, *p* = 0.022);
- for *moderate stress* (illness limiting daily activity but with retained oral intake), 48% of respondents advised extra oral GC, 9% intramuscular HC, while 43% reported no adjustment to the usual regimen; no statistically significant differences by specialty or caseload emerged in this scenario;
- for *major stress* (trauma, vomiting, or surgery preventing oral intake), 77% correctly recommended parenteral HC, while 9% suggested only additional oral GC and 14% no additional coverage; differences by specialty or caseload were not statistically significant in this setting.

### Proactive AI prevention

Overall, 82% of respondents provided family education on AI recognition and sick-day management. Education was delivered predominantly by pediatric endocrinologists (41%) or pediatric neuromuscular specialists (26%), with 15% of clinicians indicating a shared approach between both teams. Only 43% of respondents prescribed intramuscular HC and provided injection training; this practice was significantly more common among pediatric endocrinologists than pediatric neuromuscular specialists (60% vs. 20%; *p* = 0.008), and in smaller centers compared with larger centers (46% vs. 20%; *p* = 0.049).

Written emergency plans were provided by 71% of clinicians; electronic alerts within the hospital system were used in 37%, and medical alert devices recommended by 31%. Larger centers were more likely than smaller ones to encourage medical alert identification (80% vs. 23%; *p* = 0.012). Table 2 summarizes proactive measures.

**Table 2:** Proactive measures for adrenal insufficiency management in young people with Duchenne muscular dystrophy on glucocorticoid therapy**.** Values are expressed as number (percentage) of respondents. Abbreviation: HC, hydrocortisone

Measure	*n* (%)
*Family education* - Yes - No - Unsure	29 (83%) 4 (11%) 2 (6%)
*Team that provides education* - Endocrine - Neuromuscular - Both	15 (50%) 9 (30%) 6 (20%)
*Intramuscular HC prescription* - Yes - No - Unsure	15 (43%) 16 (46%) 4 (4%)
*Intramuscular injection training* - Yes - No - Unsure	15 (43%) 18 (51%) 2 (6%)
*Written emergency plan* - Yes - No - Unsure	25 (71%) 8 (23%) 2 (6%)
*Medical alert identification* - Yes - No - Unsure	11 (31%) 22 (63%) 2 (6%)
*Electronic alert in hospital system* - Yes - No - Unsure	13 (37%) 20 (57%) 2 (6%)

## Discussion

This national survey provides preliminary insight into AI prevention and management practices for children and adolescents with DMD on chronic GC therapy in Italy, revealing substantial variability across centers and specialties. Although most clinicians recognized the need for stress-dose coverage during significant illness, nearly half did not recommend any additional GC during moderate stress, and 14% still omitted parenteral HC in major stress scenarios. These gaps suggest that a considerable proportion of patients may remain insufficiently protected during acute illness, trauma, or surgery, despite clear international recommendations [1, 20–23].

The findings align with previous work showing that AI remains underrecognized in pediatric practice [12–13]. In a recent U.S. survey, only about half of families of individuals with DMD recalled discussions about AI, and fewer than one-third reported access to intramuscular HC, with emergency letters and clear action plans often lacking [26]. By contrast, a recent UK audit in two specialist centers reported that 93% of boys on GC therapy had access to emergency intramuscular HC, suggesting that near-universal preparedness is achievable in structured care settings, albeit with possible selection bias [27]. Comparable data from Italian families are not yet available, but the clinician-reported variability observed here strongly suggests system-level rather than isolated gaps.

Another key observation is the inconsistency in proactive measures such as family education, written emergency plans, emergency HC prescriptions, and use of electronic alerts or medical IDs. Although most respondents reported discussing AI with families, less than half provided intramuscular HC or practical training, and system-level safeguards (alerts in electronic health records, medical alert identification) were infrequently implemented. These patterns mirror challenges described in broader AI cohorts, where inadequate education and lack of emergency resources are associated with ongoing morbidity and mortality from adrenal crises [8–9, 12–13].

Specialty-based differences were evident: pediatric endocrinologists were more likely than neuromuscular specialists to recommend guideline-concordant stress-dose regimens and to provide intramuscular HC training, reflecting their greater exposure to AI management. Conversely, some larger centers reported lower adoption of certain proactive measures, such as injection training and medical IDs, possibly reflecting time constraints and logistical barriers in high-volume services. These findings support the need for structured, multidisciplinary pathways that clearly assign responsibilities for AI education and emergency preparedness between neuromuscular and endocrine teams [1, 20–22].

From a policy perspective, the absence of national Italian guidance on AI prevention in DMD likely contributes to practice heterogeneity. Integration of existing international frameworks—such as the updated *PJ Nicholoff Steroid Protocol* [16] and international DMD care recommendations [17]—into national protocols could promote consistent risk recognition, standardized stress-dosing algorithms, and routine provision of emergency HC and written plans [1, 20–23]. Future work should also include patient- and caregiver-focused studies in Italy to quantify awareness, preparedness, and barriers to implementing recommended AI prevention strategies, as suggested by international data [26–27].

This study has several limitations. The sample size was modest and participation was voluntary, so selection bias is possible, with more engaged or guideline-aware clinicians potentially overrepresented. Practices were self-reported rather than verified against medical records or institutional protocols, which may have led to overestimation of adherence to recommended adrenal insufficiency prevention strategies. The survey did not collect outcome data, such as adrenal crisis incidence, nor did it include patient or caregiver perspectives, limiting inferences on real-world preparedness at the family level. Finally, findings reflect the Italian healthcare context and may not be generalizable to other countries or to centers not captured in the national DMD network.

Despite these limitations, this study has several strengths. It is, to our knowledge, the first national survey specifically focused on adrenal insufficiency prevention and management in children and adolescents with Duchenne muscular dystrophy on chronic glucocorticoid therapy in Italy, including all identified centers and both pediatric endocrinologists and neuromuscular specialists. The survey combined detailed questions on glucocorticoid regimens with structured clinical stress scenarios and proactive measures (education, emergency kits, written plans, alerts), providing a granular picture of real-world practice. These features allow identification of concrete targets for harmonized protocols, multidisciplinary pathways, and educational interventions aimed at improving adrenal crisis prevention in this high-risk pediatric population.

## Conclusions

To our knowledge, this is the first national survey examining AI prevention and management practices among clinicians caring for individuals with DMD in Italy. The results suggest the presence of gaps in stress-dose coverage, family education, and emergency preparedness, despite widespread use of chronic GC therapy and clear international recommendations [1, 16–17, 20–24]. Addressing these gaps will require harmonized national guidance, closer collaboration between neuromuscular and endocrine teams, and systematic integration of AI prevention— including standard stress-dosing protocols, written emergency plans, emergency HC kits, and system-level alerts—into routine DMD care. Future research should evaluate targeted educational and organizational interventions and incorporate patient and caregiver perspectives to optimize adrenal crisis prevention and improve outcomes in this high-risk pediatric population.

## Supplementary Information

Below is the link to the electronic supplementary material.

Supplementary Material 1

## Data Availability

The data that support the findings of this study are not openly available due to reasons of sensitivity and are available from the corresponding author upon reasonable request.

## Abbreviations

AI: adrenal insufficiency
DMD: Duchenne muscular dystrophy
DFZ: deflazacort
GC: glucocorticoid(s)
HC: hydrocortisone
PDL: prednisolone
PN: prednisone
VAM: vamorolone
